# Botulinum Toxin Therapy Combined with Rehabilitation for Stroke: A Systematic Review of Effect on Motor Function

**DOI:** 10.3390/toxins11120707

**Published:** 2019-12-05

**Authors:** Takatoshi Hara, Ryo Momosaki, Masachika Niimi, Naoki Yamada, Hiroyoshi Hara, Masahiro Abo

**Affiliations:** 1 Department of Rehabilitation Medicine The Jikei University School of Medicine 3-25-8, Nishi-Shinbashi, Minato-Ku, Tokyo 105-8461, Japan; 2 Department of Rehabilitation Medicine, Teikyo University School of Medicine University Hospital, Mizonokuchi, Kanagawa 213-8507, Japan; 3 Rehabilitation Center, Ainomiyako Neurosurgery Hospital, Osaka 538-0044, Japan

**Keywords:** stroke, botulinum toxin, spasticity, rehabilitation, upper limbs, lower limbs, motor function

## Abstract

Aim: The purpose of this study was to examine the effectiveness of botulinum toxin A (BoNT-A) therapy combined with rehabilitation on motor function in post-stroke patients. Methods: The following sources up to December 31, 2018, were searched from inception for articles in English: Pubmed, Scopus, CINAHL, Embase, PsycINFO, and CENTRAL. Trials using injections of BoNT-A for upper and lower limb rehabilitation were examined. We excluded studies that were not performed for rehabilitation or were not evaluated for motor function. Results: Twenty-six studies were included. In addition to rehabilitation, nine studies used adjuvant treatment to improve spasticity or improve motor function. In the upper limbs, two of 14 articles indicated that significant improvement in upper limb motor function was observed compared to the control group. In the lower limbs, seven of 14 articles indicated that significant improvement in lower limb motor function was observed compared to the control group. Conclusions: The effect of combined with rehabilitation is limited after stroke, and there is not sufficient evidence, but results suggest that BoNT-A may help to improve motor function. In future studies, the establishment of optimal rehabilitation and evaluation times of BoNT-A treatment will be necessary for improving motor function and spasticity.

## 1. Introduction

Post-stroke patients with upper and lower limb hemiparesis may present with spasticity, which is a symptom of upper motor neuron syndrome [1]. Previous reports have indicated that spasticity is observed in 19% of patients at three months following stroke and in 38% of patients at 12 months following stroke [2,3]. Spasticity occurs after stroke in between 18% and 38% of patients and may interfere with the execution of daily activities, social participation, and quality of life [4]. Spasticity can interfere with the functional recovery of upper limbs, especially actions such as raising arms, the opening and closing of hand and fingers, and holding objects [4]. Therefore, patients may have difficulty maintaining cleanliness and in eating and dressing activities. In lower limbs, spasticity primarily affects walking. An ample range of motion (ROM) and strength is required for walking, and spasticity makes it difficult to adjust the ROM and control muscle contractions [5]. In particular, the continuous contraction of the triceps surae muscle can lead to clonus, which, in turn, may result in an equinovarus foot [5]. An equinovarus foot can result in poor toe clearance during the swing phase of gait and ankle instability during weight bearing [6]. Botulinum toxin A therapy (BoNT-A) temporarily reduces muscle activity by preventing the release of acetylcholine at the neuromuscular injection, resulting in a reduced spasticity and muscle tone [7]. The pharmacological effect of an intramuscular injection of botulinum toxin type A commences at two-to-four days following injection, with an expected peak effect at three weeks, and its efficacy persists six weeks after injection and up to nine and twelve weeks [8,9]. Several open and placebo-controlled studies have reported the efficacy of local botulinum toxin injections in reducing spasticity and have emphasized its ease of use and safety [9,10,11,12]. Recently, there have been reports about the improvement in motor function for post-stroke hemiparesis using BoNT-A [13]. It has been suggested that further improvement of motor function can be expected when using BoNT-A combined with rehabilitation. In an international survey, Bakheit indicated that overall rehabilitation is likely to be more important in producing functional change than a single specific intervention, such as BoNT-A injection [14]. However, no systematic review has examined changes in motor function by BoNT-A combined with rehabilitation, and there have been few reports that have focused on motor function. Recently, we reported a combined treatment program of BoNT-A therapy with multidisciplinary rehabilitation and suggested that this combined treatment was effective for the improvement of motor function in post-stroke patients with upper and lower limb spasticity [15]. In addition, in the lower extremities, these effects are associated with the degree of muscle fibrosis [16]. Furthermore, repeated BoNT-A therapy and rehabilitation may modify not only the lower limb motor function and walking speed but also changes in bracing [17].

The purpose of this study was to review the literature on improvement of upper and lower limb motor function by BoNT-A combined with rehabilitation and to investigate the future direction of research in this field.

## 2. Results

### 2.1. Study Selection

**Figure 1:**
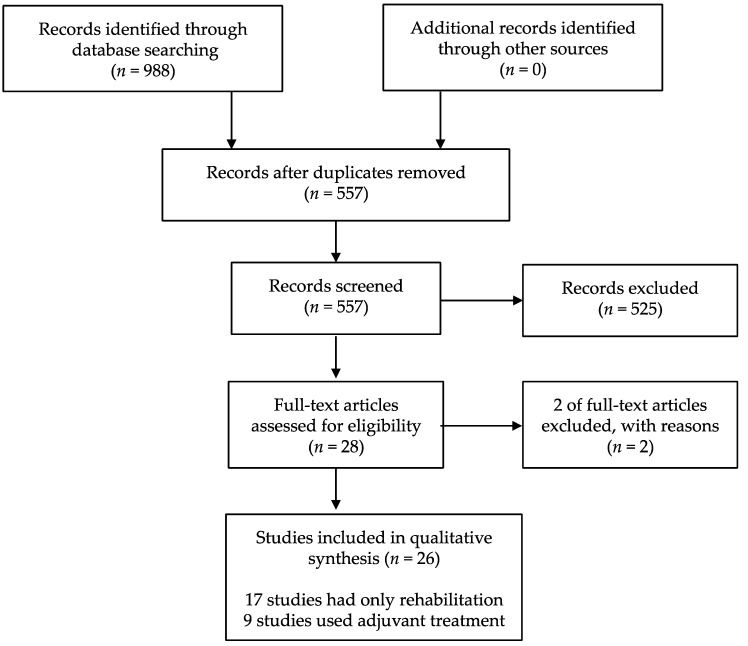
Study flow diagram.

After screening 988 citations, 28 potentially relevant studies were identified. After review, 26 articles met the predetermined inclusion criteria (Figure 1). The subjects data and result summary of each study are given in Table 1 and Table 2 and noted below [18,19,20,21,22,23,24,25,26,27,28,29,30,31,32,33,34,35,36,37,38,39,40,41,42,43,44,45]. Two of the included articles used data from previously published studies. Therefore, these studies were regarded as one article [30,31,38,39].

### 2.2. Description of Studies

All trials that were selected for this review were published up to December 2018 and were in English. The 26 studies included a total of 1307 patients that received BoNT-A and rehabilitation therapy with a sample size varying from 15 to 332 patients. Twenty-three articles of this review were randomized controlled trials (RCTs) (two article were cross-over trials). In two of these trials, repeat BoNT-A was performed during the intervention [30,31,35]. In addition to rehabilitation, nine studies used adjuvant treatment to improve spasticity or motor function. There were 12 trials for the upper limbs, 12 trials for the lower limbs, and two trials for the upper and lower limbs. The mean age range of the intervention group was 43.6–67 years, and the mean age range of the control group was 41.2–66.0 years [18,19,20,21,22,23,24,25,26,27,28,29,30,31,32,33,34,35,36,37,38,39,40,41,42,43,44,45]. The average time between onset and treatment was 24.2 days to 15.7 years (the units of one study was unclear). In the control group, four trials were of BoNT-A only, seven trials were of placebo injection, and nine trials were of rehabilitation only (robotic training alone was included). Uchiyama et al. (2018) used Group 1 as BoNT-A combined with physical therapy (PT) and occupation therapy (OT), and Group 2 used the first phase as rehabilitation only and the second phase as BoNT-A combined with PT and OT [18]. Pimentel et al. (2014) compared BoNT-A doses at 300 and 1000 U [26]. Lim et al. (2008) administered BoNT-A for shoulder pain and spasticity and compared it to triamcinolone acetonide [33]. Of the studies in which adjuvant treatment was used in combination, three trials were BoNT-A combined with rehabilitation in the control group [37,40,45]. The list of control groups in each study is shown in Table 3.

### 2.3. Risk of Bias Assessment

Methodological quality by risk of bias is shown in Table 4 [18,19,20,21,22,23,24,25,26,27,28,29,30,31,32,33,34,35,36,37,38,39,40,41,42,43,44,45]. Sequence generation was appropriate in 57.6% of the studies. In allocation concealment, 38.4% of the studies were appropriate. In regard to the blinding of participants and personnel, some non-RCT studies were marked high risk [18,25,36]. In addition, in the case where the control group was not placebo injection, it was considered difficult to blind the subjects because the intervention content differed between the intervention group and the control group. Therefore, we marked high risk. In regard to the blinding of outcome assessment, the evaluator was properly blinded in 65.3% of the studies. Regarding the outcome data, many studies properly described the dropout and the completion of trial (88.4%). Regarding selective reporting, the study with no comparison between groups and the control group and the study that indicated the change in the results were marked high risk. In addition, in regard to the injection of BoNT-A, the study without a description of the injection site and injection volume and the study without a description of rehabilitation implementation time were regarded as high risk.

### 2.4. Outcome Measure

**Figure 2:**
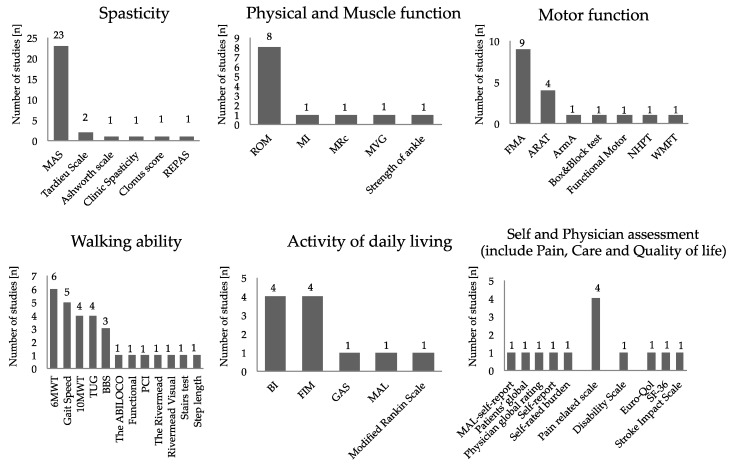
Overview of outcome measure: The vertical axis represents the number of studies. The horizontal axis represents the evaluation methods that used in each study. Abbreviations: 10MWT, 10 m walk test; 6MWT, six minute walk test; ARAT, Action Research Arm Test; ArmA, the arm activity measure; BBS, Berg Balance Scale; BI, Barthel Index; FIM, Functional Independence Measure; FMA, Fugl–Meyer Assessment; GAS, goal attainment scaling; MAL, the motor activity log; MAS, Modified Ashworth Scale; MI, Motricity Index; MRc, the medical research council scale; MVG, maximum voluntary grip strength; NHPT, Nine Hole Peg Test; PCI, physiological cost index; REPAS, resistance to passive movement Scale; ROM, range of motion; SF-36, MOS 36-Item Short-Form Health Survey; TUG, Timed Up and Go Test; and WMFT, Wolf Motor Function Test.

**Figure 3:**
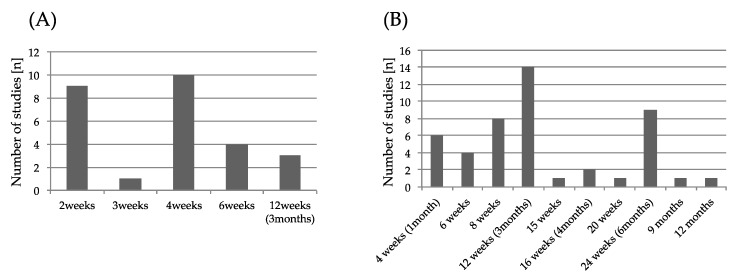
(**A**) Initial evaluation timing after botulinum toxin A (BoNT-A) injection. (**B**) Second and subsequent evaluation timing. The vertical axis represents the number of studies. The horizontal axis represents the evaluation timing after BoNT-A injection.

An overview of assessments of outcomes measure in the intervention is shown in Figure 2. For the assessment of spasticity, the Modified Ashworth Scale (MAS) was the most common. As for motor function evaluation, the Fugl–Meyer Assessment (FMA) was the most adopted for the evaluation of spasticity and motor function evaluation, of which five cases were for upper limb function and two were for lower limb function. The next most adopted was the Action Research Arm Test (ARAT). Regarding walking ability, the six minute walk test (6MWT) was adopted most, followed by walking speed and balance evaluation. The Barthel Index (BI) and the Functional Independence Measure (FIM) were frequently adopted for the activity of daily living. Regarding follow-up, 80.7% of the studies were evaluated at multiple times. Follow-up was evaluated for a minimum of two weeks (26.9%) and a maximum of 120 days after administration. The timing of the first evaluation after intervention and second and subsequent evaluation timing after intervention during follow up are shown in Figure 3. The timing of the first evaluation was most often after four weeks and secondly at two weeks. The timing of evaluation after the first follow-up concentrated on evaluation within 12 weeks and at 24 weeks.

### 2.5. Intervention in BoNT-A Therapy

The dose of BoNT-A was the applied dose for each drug. Guided dosing with electromyography (EMG), electrical stimulation (ES), or ultrasonography (US) was performed in 57.6% of the studies. As mentioned above, in two studies, multiple doses were administered in compliance with the dosing interval [30,31,35]. For some studies, although there was mention of botulinum toxin, there was no mention of the dose volume or location or both (30.7%) [19,21,22,23,30,31,32,40,42].

### 2.6. Rehabilitation

In many studies, the contents of rehabilitation, rehabilitation time, units per day or week, and the total number of sessions were described. The most described training was stretch (42.3%). After that, range of motion (19.2%) and gait training (19.2%) were described frequently. Total sessions varied from study to study and ranged from five to 120 sessions. There were three studies that used ES or functional electrical stimulation (FES) as adjuvant treatment, three studies that used a robot, two studies that did taping and casting, and one study that used constraint-induced movement therapy (CIMT) [36,37,38,39,40,41,42,43,44,45].

### 2.7. Effect of BoNT-A and Rehabilitation of Motor Function

First, the intervention group had improved spasticity in 23 of 24 studies, except for two studies that did not evaluate spasticity. However, there was no significant difference in improvement in spasticity when compared to the group in which the control group was BoNT-A alone [21,22,36,41]. Moreover, in the studies compared with the group where the control group was placebo injection, three of seven studies were compared among the groups and were considered to have a significant difference [24,27,34]. However, there was also a report showing no significant improvement compared with placebo injection in the comparison between groups [32]. In comparison to triamcinolone acetonide, the MAS did not improve significantly compared to the control group [33]. In regard to adjuvant treatment, in studies where the control group was BoNT-A and regular rehabilitation, there was no significant difference in spasticity among the groups compared [40,45].

Regarding motor function related to the upper extremity, two of the 14 articles that treated the upper extremities indicated that significant improvement in upper extremity function was observed compared to the control group [21,45]. Devier et al. (2017) reported a significant improvement in the FMA compared to the control group (BoNT-A only) after 18–21 and 24–27 weeks [21]. Sun et al. (2010) reported that there was a significant improvement in the ARAT compared to the control group (BoNT-A plus rehabilitation) at three or six months after the intervention [45]. In addition, the motor activity log (MAL) also reported that there was a significant improvement after three or six months. On the other hand, nine of 14 studies showed improvement in upper limb function by the intervention, but there was no significant difference or comparison with the control group [19,20,28,29,30,31,32,37,42]. Wolf et al. (2012) reported that there were no group-by-time interactions for changes in the Wolf Motor Function Test and no treatment difference, although the intervention group could complete more tasks governing proximal joint motions [29]. Meythaler et al. (2009) reported that, compared to the control group (placebo injection), the intervention group showed a slightly enhanced functional status of stroke subjects beyond that obtained with therapy alone at 12 weeks after injection [32]. Suputtitada et al. (2005) indicated a significant decrease in ARAT results at week eight compared with control (placebo injection) in the 1000 U group, but the 500 U group showed improved ARAT results, which peaked at week eight compared to the control group [34]. Pennati et al. (2015) indicated improvement in upper limb function in both groups, but both the FMA and Box and Block test showed better improvement in the control group (rehabilitation only) [42]. In three of 14 studies, there was no significant improvement by intervention [26,27,30,31]. In particular, Demetrios et al. (2014) mentioned that there were no significant differences in change scores for the assessment of upper limbs [25].

Regarding motor function related to the upper extremity, seven of 14 studies that administered BoNT-A to the lower extremity indicated that significant improvement in motor function was observed compared to the control group [22,23,24,36,39,41,43]. Tao et al. (2015) reported that there was a significant difference in step length, cadence, and speed at eight weeks, as well as the FMA and 6MWT compared to the control group (placebo injection) [24]. Fujita et al. (2019) reported that, as compared with the control group (BoNT-A only), walking speed and cadence were significantly increased and the rate of change was significantly greater [36]. In addition, in the intervention group, the step length increased significantly, and an improvement of the asymmetry index of step length was observed. On the other hand, one participant in the intervention group and seven participants in the control group indicated that their walking speed decreased. Johnson et al. (2002, 2004) reported that the physiological cost index (PCI) decreased slightly in the intervention group with improvement in lower extremity function, which was not observed in the control group (rehabilitation only) [38,39]. Though five studies showed improvement in motor function by intervention, there was no significant difference compared with control group or there was no comparison between groups [18,19,26,35,40]. Interestingly, Burbaud et al. (1996) focused not only on changes in lower limb motor function but also on changes in aids. They reported that two patients in the intervention group who previously could not walk independently were able to walk alone with an ordinary walking stick, two patients were able to exchange their tripod stick for an ordinary walking stick, and two other patients no longer needed walking sticks [35]. On the other hand, in two of 14 studies, there was no significant difference between the groups regarding lower limb motor function [25,44].

## 3. Discussion

This review focused on examining the effectiveness of BoNT-A treatment combined with rehabilitation on motor function in post-stroke patients. Twenty-six studies were reviewed, including 1307 patients. There was a significant effect observed in 23 studies in the intervention group on spasticity [18,19,20,21,22,24,25,26,27,28,29,30,31,32,34,35,36,37,40,41,42,43,44,45]. In regard to upper limb motor function, 11 of 14 cases showed improvement, but two studies showed significant differences in comparison with the control group [19,20,21,25,27,28,29,30,31,32,33,34,37,42,45]. In regard to lower extremity motor function, 12 of 14 cases showed improvement, and seven studies showed a significant difference in comparison to the control group [18,19,22,23,24,25,26,35,36,38,39,40,41,43,44].

In regard to study design, the control group was set to various types of studies, because many studies did not focus on the improvement of motor function. Previous reports have shown that BoNT-A administration is effective and safe against spasticity [9,10]. Therefore, in studies focusing on the improvement of motor function, setting the control group as placebo injection may not be effective.

In regard to evaluation, both upper and lower limbs were widely used for motor function evaluation in rehabilitation. For the upper limbs, Wolf et al. (2012) mentioned the possibility of proximal improvement. Takekawa et al. (2013) reported improvement in upper extremity function by combining home-based functional training with past BoNT-A injection [46]. Among these, categories A and B improved significantly after one, three, and six months in evaluation by the FMA, and category D improved significantly at three and six months. An improvement of motor function by BoNT-A and rehabilitation may start from the proximal side [46]. Therefore, it may be necessary to carefully observe changes in each category, as well as the total score of motor function evaluation of upper limb function, in order to capture the improvement effect for the upper limbs. For the lower limbs, some studies focused on walking speed and walking durability. In fact, our past study reported that we observed not only improved walking speed but also improved balance using the Timed Up and Go Test (TUG) [15]. According to a review of balance assessment after BoNT-A injection by Phadke et al. (2014), the evidence for balance changes after BoNT-A is weak because of a lack of randomization, control group comparison, objective balance assessment measures, and standard clinical scales [47]. Therefore, in regard to balance, a new systematic review is required in the future. Burbaud et al. (1996) reported not only a change in lower limb motor function but also a change in the aid and reported improvement [35]. A systematic review of gait velocity in randomized controlled trials reported a 0.044 m/s increase (an effect size of 0.193) in gait velocity in the treatment groups, although the number of studies reporting such an improvement was small [48]. In our previous study, we found that the home ambulation group (gait speed < 0.4 m/s) demonstrated a significant change in 10 M gait velocity compared to the limited community ambulation (gait speed = 0.4–0.8 m/s) and full community ambulation (gait speed > 0.8 m/s) groups [15]. Conversely, the full community ambulation group showed a ceiling effect on the post-intervention change. Therefore, there is a limit to the evaluation of improvement of the lower limbs in regard to walking speed only. These results suggest that a multifaceted evaluation focusing on an evaluation classified according to walking speed before intervention, a balance index, and a change of aid is necessary.

Due to the pharmacological activity of BoNT-A, the follow-up period was evaluated at one month and three months, which is the most effective time and the time when the effect disappears, respectively. However, some studies reported a temporary loss of function due to the loss of muscle strength associated with the relief of spasticity by BoNT-A [34,36]. Therefore, multiple evaluations are important in order to grasp the timing when temporary functional decline occurs and the timing when functional improvement is most recognized.

In regard to BoNT-A injection, many studies used methods guided by EMG, ES, or US [20,21,22,23,24,28,33,34,35,36,38,39,40,41,42,43,44]. It has been suggested in past studies that the guided injection method is safer and more accurate than administration by anatomical landmarks, which is usually recommended [49]. BoNT-A also has localized and dose-dependent effects on the injected muscle [50,51]. Therefore, it is necessary to describe the total volume, the dosage volume to each muscle, and the dosage method in detail. Regarding rehabilitation and BoNT-A treatment, a detailed description is required. In this review, the frequency and load of rehabilitation varied. Additionally, it cannot be said that frequent rehabilitation interventions always contribute to the improvement of motor function. Only stretch was shown to be effective against spasticity from past reports [52]. There were also reports of adjuvant treatment, but there were few reports focusing on motor function [36,37,38,39,40,41,42,43,44,45]. In the future, it is necessary to report not only on spasticity with adjuvant treatment but also on the improvement of motor function.

In regard to the risk of bias, the quality of reporting was poor. Though RCTs were used in some studies, there was limited mention of randomization methods. Additionally, for the evaluation, there were insufficient blinds. Because BoNT-A was combined with rehabilitation in this review, both blinding studies were required for the recommended studies. However, many studies were aimed mainly at the effect on spasticity, so few studies were both blinded. In addition, blinding may be difficult at the time of the set up of study design. Regarding the analysis of significant differences in results, some studies did not adequately compare with the control group.

From the above findings, the following items are recommended for conducting future research on the effects of motor function by BoNT-A and rehabilitation:

(i) It is known that BoNT-A is effective for spasticity. Therefore, BoNT-A alone or other rehabilitation is desirable for the control group.

(ii) In the evaluation of upper extremity function, it is recommended to focus not only on the total score of the evaluation but also on changes in sub-score.

(iii) In the evaluation of lower limb function, it is recommended to focus not only on the walking speed but also on changes in balance, changes in walking patterns, and changes in aids.

(iv) It is recommended to follow-up more than once in order to catch the temporary functional decline and the timing that the function improved most.

(v) For the injection of BoNT-A, the guided method is recommended. In addition, it is desirable to describe injection the volume and injection muscle.

(vi) It is desirable to describe details, as much as possible, about the rehabilitation (the time of day, the number of times of week, the total number of times, etc.).

## 4. Conclusions and Future Perspective

The effect of BoNT-A combined with rehabilitation is limited after stroke and there is not sufficient evidence, but it has been suggested that BoNT-A may help improve motor function. In the future, the establishment of an optimal rehabilitation and the optimal evaluation time in BoNT-A treatment will be a breakthrough in improving both motor function and spasticity.

## 5. Methods

### 5.1. Criteria for Considering Studies for Review

#### 5.1.1. Type of Studies

Randomized controlled trials (RCT), non-RCT, cross-over studies, and comparative studies were included in this analysis. Protocols, retrospective studies, and case reports were excluded. Abstracts and non-English language publications were also excluded.

#### 5.1.2. Type of Participants

We included patients with upper and lower limb spasticity after stroke. Patients were >18 years old. Publications included >50% of patients with stroke and 3 or more patients in the cohort.

#### 5.1.3. Type of Interventions and Comparisons

We included studies that performed BoNT-A injection in the upper or lower limbs, with rehabilitation performed after injection, and with assessment of motor function. We excluded studies that did not perform rehabilitation or did not evaluate motor function.

#### 5.1.4. Search Strategy

Searches were performed on the following publication databases: Pubmed, Scopus, CINAHL, Embase, PsycINFO, and CENTRAL. Studies published in English and on or before 31 December 2018 were selected. Selected keywords included stroke, cerebral vascular accident, ischemic stroke, hemorrhagic stroke, botulinum toxin, botulinum toxin therapy, antispastic therapy, rehabilitation, physical therapy, occupational therapy, intensive rehabilitation, multidisciplinary rehabilitation, motor, function, ability, walk, and capacity. Variations of keywords were individualized for each scientific database. The references of all retrieved articles were reviewed to ensure that all relevant articles were included for data synthesis. As an example, the Pubmed search strategy is illustrated in Appendix A.

### 5.2. Date Collection and Analysis

#### 5.2.1. Selection of Studies

Two authors (T.H. and R.M.) independently reviewed all potential studies for inclusion against the eligibility criteria. They examined the title and abstract, and, where necessary, the full text of studies to assess if they were eligible for inclusion. If they could not reach agreement by discussion, a third author (M.N.) made the final decision concerning eligibility.

#### 5.2.2. Date Extraction

Two authors (T.H. and R.M.) independently used a standard form to extract study characteristics and outcome data from the studies. Discrepancies were checked against the original data. A third author (M.N.) made the final decision in the cases of disagreement.

#### 5.2.3. Assessment of Risk of Bias in the Included Studies

We assessed the methodological quality of selected studies as described in the Cochrane Review Groups [53]. We created a risk of bias table and included description and judgment (low risk of bias, high risk of bias, or unclear risk of bias) for the following domains for each of the included studies: (1) random sequence generation, which is selection bias (biased allocation to interventions) due to the inadequate generation of a randomized sequence; (2) allocation concealment, which is selection bias (biased allocation to interventions) due to the inadequate concealment of allocation prior to assignment; (3) the blinding of participants and personnel, which is performance bias due to knowledge of the allocated interventions by participants and personnel during the study; (4) the blinding of outcome assessment, which is detection bias due to knowledge of the allocated interventions by outcome assessors; (5) incomplete outcome data, which is attrition bias due to the amount, nature or handling of incomplete outcome data; (6) selective reporting, which reporting bias due to selective outcome reporting; and (7) other sources of bias, which are considered bias due to problems not covered elsewhere in the table. Two review authors independently performed quality assessment. Any disagreement between authors arising at any stage was resolved through discussion or through a third author.

Because many trials concerning the effect of BoNT-A for stroke patients have focused on the effect of spasticity and few studies have focused on motor function, in this review, a meta-analysis was not performed. This study was prospectively registered with the PROSPERO database of systematic reviews (CRD42019132145).

## Figures and Tables

**Table 1:** Study and subject characteristics.

Study	Country	Limb	Design	Sample	Sex(M:F)	Age	Time Between Onset and Treatment
Combined Rehabilitation							
Uchiyama Y et al. 2018 [18]	Japan	Lower	Comparative study	Group1:9(BoNT-A combined PT and OT) Group2:10(Group2:First Phase: PT, OT Second Phase BoNT-A combined PT and OT)	15:4	Group1 57.0 (51.0–65.5), Group2:58.5 (47.0–65.6)	Group1 17.0 (11.5–39.0), Group2:35.5 (18.5–105.3) month
Prazeres A et al. 2018 [19]	Brazil	Both	RCT (*vs Placebo injection*)	I:20 C:20	24:16	I:52.5(11.0) C:52.5(12.5)	I:34.1(21.4) C:32.1(14.9) months
Umar et al. 2018 [20]	Pakistan	Upper for focal dystonia	RCT (*vs rehabilitation only*)	I:23 C:23	26:17	I:43.6(10.9) C:48.8(10.8)	NR
Devier et al. 2017 [21]	USA	Upper	RCT (*vs BoNT-A only*)	I:15 C:16	21:10	I:58.0(6.6) C:60.9(11.0)	6(0.5–16.5) years
Roche et al. 2015 [22]	France	Lower	RCT (*vs BoNT-A only*)	I:19 C:16	25:10	I:47.8(14.4) C:51.5(13.5)	I:15.7(6.9) C:7.3(3.6) years
Ding et al. 2015 [23]	China	Lower	RCT *(vs rehabilitation only)*	I:33(BoNT-A, AFO, Conventional therapy(Co), rehabilitation) Observation:35(BoNT-A, Co, rehabilitation) C:35(Co, rehabilitation)	49:54	I:63.4(10.2) Observation:62.8(11.5) C:64.2(12.4)	I:17.0(1.1) Observation:16.4(1.2) C:15.4(1.8) ?
Tao et al. 2015 [24]	China	Lower	RCT *(vs Placebo injection)*	I:11 C:12	15:8	I:55(12) C:58(14)	I:24.2(12.2) C:23.2(17.2) days
Demetrios et al. 2014 [25]	Australia	Both (Upper 40 Lower 37)	Comparative study *(vs rehabilitation only)*	I:28(BoNT-A, Standard Care) C:31 (Placebo, Standard Care)	42:17	I:60.6(48.6–65.9) C:61.4(47.8–68.6)	I:2.3(1.1–5.5) C:2.5(1.1–5.0) years
Pimentel et al. 2014 [26]	Brazil	Lower	RCT *(300U vs 100U)*	First group(300U):11 Second group(100U):12	10:11	First group:50.5(6.8) Second group:47.9(3.8)	First group:41.6(63.4) Second group:34.5(33.8) months
Rosales et al. 2012 [27]	Philippines	Upper	RCT *(vs Placebo injection)*	I:83 C:80	109:54	I:55.7(23–79) C:54.5(17–79)	I:7.7(3.1) C:7(2.9) weeks
Hesse et al. 2012 [28]	Germany	Upper	RCT *(vs rehabilitation only)*	I:9 C:9	6:12	I:57(11) C:66(11)	I:5.8(1.3) C:5.6(1.1) weeks
Wolf et al. 2012 [29]	USA	Upper	RCT *(vs Placebo injection)*	I:13 C:12	15:10	I:48.8(15.6) C:49.8(13.7)	NR
Shaw et al. 2011, 2010 [30,31]	UK	Upper	RCT *(vs rehabilitation only)*	I:170 C:162	225:107	I:67(58.8–74) C:66(59.8–72.3)	I:324(128.5–1387.5) C:280(148.8–1145.8) days
Meythaler et al. 2009 [32]	USA	Upper	RCT with cross-over trial (12 weeks) *(vs Placebo injection)*	21	15:6	53.3(14.8)	more than 6 months
Lim et al. 2008 [33]	Korea	Upper	RCT *(vs Triamcinolone acetonide)*	I:16 C:13	15:14	I:64.8(2.1) C:57.1(3.6)	I:230.4(53.8) C:299.5(73.9) days
Suputtitada et al. 2005 [34]	Thailand	Upper	RCT *(vs Placebo injection)*	I:45(350U:15,500U:15,1000U:5) C:15	26:24	350U:46.5(8.5),500U:53(18.7),1000U:59.9(9.2) C:55.2(8.9)	350U:7.9(0.9),500U:8.4(0.7),1000U:8.7(0.4) C:8.5(0.8) months
Burbaud et al. 1996 [35]	France	Lower	RCT with cross-over trial(90days) *(vs Placebo injection)*	I:10 C:13	16:7	I:50.7(11) C:53.9(16)	I:23.2(36) C:23.8(33) months
Combined ES, FES and rehabilitation							
Fujita et al. 2018 [36]	Japan	Lower	Non-RCT *(vs BoNT-A only)*	I:17(ES) C:17	25:9	I:58.6(10.5) C:57.2(10.5)	I:39.8(37.7) C:75.2(51.2) months
Weber et al. 2010 [37]	USA	Upper	RCT *(vs BoNT-A + rehabilitation)*	I:10(BoNT-A FES Rehabilitation) C:13(Rehabilitation)	8:15	I:54.0(10.3) C:41.2(14.2)	I:9.7(8.6) C:4.3(2.5) years
Johnson et al. 2002, 2004 [38,39]	UK	Lower	RCT *(vs rehabilitation only)*	I:10(BoNT-A FES Rehabilitation) C:8(Rehabilitation)	12:6	I:59.3(12.5) C:58.2(12.7)	0–6 months:9, 6–12 months:9
Combined Robot and rehabilitation							
Erbil et al. 2018 [40]	Turkey	Lower	RCT *(vs BoNT-A + rehabilitation)*	I:32(BoNT-A, RAT and physical therapy) C:16(BoNT-A, physical therapy)	27:16	I:50.1(11.8) C:48.7(10.4)	I:39(34.3) C:25.9(24.6) months
Picelli et al. 2016 [41]	Italy	Lower	RCT *(vs BoNT-A only)*	I:11(BoNT-A, RAGT) C:11(BoNT-A)	16:6	I:62.4(9.5) C:65.1(3.4)	I:6.2(4.2) C:6.1(3.8) years
Pennati et al. 2015 [42]	Italy	Upper	RCT *(vs rehabilitation only)*	I:7(BoNT-A, robotic training) C:8(robotic training alone)	9:6	53.66(38–69)	10 months–20 years
Combined Taping, Casting and rehabilitation							
Carda S et al. 2011 [43]	Italy	Lower	RCT *(vs rehabilitation only)*	Taping:24, Casting:27, Stretching: 18	35: 34	Taping:62.2(11.7), Casting:64.5(12.5), Stretching:59.6(14.3)	Taping:46.9(41.3), Casting:52.3(43.8), Stretching:43.9(39.6) months
Karadag-Saygi E et al. 2010 [44]	Turkey	Lower	RCT *(vs rehabilitation only)*	I:10(BoNT-A, kinesiotaping) C:10	12:8	I:63.8(9) C:57.3(12)	I:35.2(29) C:39.4(30) months
Combined CIMT							
Sun et al. 2010 [45]	Taiwan	Upper	RCT *(vs BoNT-A + rehabilitation)*	I:15(BoNT-A, CIMT) C:14(BoNT-A, convention rehabilitation)	24:5	I:58.7(9.9) C:61.5(9.4)	I:2.9(1.5) C:2.9(1.3) years

Abbreviations: AFO, ankle–foot orthosis; BoNT-A, botulinum toxin A; C, control; Co, conventional therapy; I, intervention; NR, no report; OT, occupation therapy; PT, physical therapy; RCT, randomized controlled trial; CIMT, constraint-induced movement therapy; ES, electrical stimulation; FES, functional electrical stimulation; I, intervention; RAGT, robot-assisted gait training; and RAT, robot-assisted training.

**Table 2:** Intervention protocol and result summary.

Study	BoNT-A-Dosage-Location	Rehabilitation Protocol	Assessments	Follow-up	Results
Combined Rehabilitation					
Uchiyama Y et al. 2018 [18]	BotoxⓇ 300U -Gastrocnemius, Soleus, TP, FDL	1-h physical therapy and a 1-h occupational therapy for a total of 2 h/d and 5 d/wk.Total 4 weeks, ROM, Stretch, Gait training, Endurance training	MAS, ROM, Gait Speed, 6MWT, TUG, BBS	Group1: 4 week Group2: 4,8 week	Gait speed, 6MD, and TUG scores improved in group II. Intergroup comparisons at week 4 showed significantly greater improvements in the MAS score of ankle plantar flexor, ROM of ankle dorsiflexion, and 6MD in group I than in group II
Prazeres A et al. 2018 [19]	DysportⓇ -Dosage and location had no data.	30minutes twice/week, Stretch, mobilization, flexibility, endurance and functional training.	MAS, FMA, 6MWT, TUG	3, 6, 9 months	MAS was a significant tonus decrease in elbow flexors and wrist flexors in BoNT-A group. Motor function was significantly improved after 6 months in both group. TUG and 6MWT was improved after third month in the both groups. But, there was no difference between groups during follow-up.
Umar et al. 2018 [20]	DysportⓇ by EMG 350-500U - B, BB, TB, FDS, FDP, FCU, FCR, EPL, FPL	The task-specific training at one week after administration of the injections, the duration of one hour, at a frequency of three times a week for a total of 8 weeks	MAS, FMA	4,8 weeks	Both groups showed significant improvements on MAS and FMA. no significant differences were observed between the groups at baseline, after 4 and 8 weeks of intervention.
Devier et al. 2017 [21]	OnabotulinumtoxinA by EMG. -Dosage and location had no data.	24 weekly rehabilitation program. 1.5 h PT and OT included techniques such as electrical stimulation and 1h daily home exercise program between visits.	MAS, FMA, FIM, Self-report	6, 12, 15, 18–21, 24–27 weeks	Both groups had a reduction in spasticity following injection. Intervention group was significantly improved on the Fugl–Meyer upper extremity score
Roche et al. 2015 [22]	BotoxⓇ by ES - Gluteus magnus, RF, Crurails, Ham, Soleus, Calf muscles, FDB, FDL -Dosage had no data.	a standardized home-based self-rehabilitation program that consisted of 3 parts (10 min each). 4 weeks. Stretch, task-oriented exercise.	MAS, the ABILOCO Scale, 10MWT, 6MWT, TUG, MRc, Stairs test	1 month	Intervention group was significantly improved in gait speed, 6MWT, Stairs test
Ding et al. 2015 [23]	BoNT-A by US -Dosage and location had no data.	Bobath concept, ROM, walking, massage, ADL training. Duration and frequency was not reported	CSI, FMA, BBS, FIM	1, 3, 6 months	Intervention and Observation group was significantly improved after 1 month in CSI, BBS, FMA, FIM. Intervention group was significantly improved after 3 and 6 months compared other groups in CSI, BBS, FMA, FIM.
Tao et al. 2015 [24]	BoNT-A by ES 200U-Gastrocnemius, Soleus, TP	Gait training, the neurodevelopmental technique and motor relearning program physiotherapy (45 min every workday) and occupational therapy (30 min every workday).	MAS, FMA, 6MWT, modifiedBI	4,8 weeks	The gait analysis, FMA, and MBI results in Intervention group were better than those in control group.
Demetrios et al. 2014 [25]	DysportⓇ:54 patients, BotoxⓇ:5 patients mean dose I:766(244), C:673(314), -pectoralis, latissimus dorsi, corachobrachialis, B, BB, BR, PrT, pronator quadratus, FCU, FCR, FDS, FDP, FPL, adductor pollicus, flexor pollicus brevis, opponens pollicus, vastus intermedius, RF, gastrocnemius medial, gastrocnemius lateral, soleus, TP, tibialis anterior, flexor hallicus longus, flexor hallicus brevis, FDB, FDL	I: 3 or more 1-h sessions per week for approximately 10 weeks C: ≤2 × 1-h sessions per week. All participants received goal-directed, individualized rehabilitation programs, consistent with ‘real-life’ rehabilitation practices. motor learning, strengthening, postural awareness, balance training, aerobic/ conditioning exercises, range of movement, stretching, adaptive/ com- pensatory strategies (environmental adaptation, one handed skills), task specific practice and sensory training.	MAS, ArmA, 10MWT, GAS, Self-rated burden	6, 12, 24 weeks	Intervention group showed greater reduction in MAS score compared with control group at 6 and 12 weeks. Upper limb function and Gait speed was not significantly changed after Intervention and between group.Both groups showed significant improvement in goal attainment and participant satisfaction up to 24 weeks.
Combined Rehabilitation					
Rosales et al. 2012 [27]	DysportⓇ 500U -BB, BR, FCU, FCR, FDS, FDP, FPL	All patients continued with their standard rehabilitation programs throughout the study, as deemed suitable by the attending physician. These generally consisted of a 30- to 60-min program of range of motion plus stretching exercises, strengthening and endurance exercises, and electrical stimulation in some cases.	MAS, ROM, Modified Rankin Scale scores, Functional Motor Assessment Scale, VAS-pain, BI	2, 4, 8, 12, 24 weeks	Intervention group was significantly improved in MAS. Motor function was not significantly improved.
Hesse et al. 2012 [28]	XeominⓇ by US 150U - FDP, FDS, FCR, FCU	Comprehensive rehabilitation in both groups. The multiprofessional motor rehabilitation program was identical in both groups. It included physiotherapy (45 min every workday) and occupational therapy (30 min every workday); speech therapy, neuropsychology and spa therapy	MAS, REPAS, FMA, Disability Scale	4, 6 weeks	MAS, REPAS and Disability Scale was improved after4 weeks, FMA was not improved.
Wolf et al. 2012 [29]	BotoxⓇ MAX300U -wrist and finger muscles	One-hour session divided into 3 sessions. That sessions were scheduled per week beginning approximately 1 month after injections and continued until 12 to 16 treatment sessions were completed	MAS, ROM, WMFT, Stroke Impact Scale	1, 2, 3 months	MAS scores improved for the BTX-A group and worsened for the control group after injection. There were no group-by-time interactions for changes in the WMFT and no treatment difference, although the Intervention group could complete more tasks governing proximal joint motions.
Shaw et al. 2011, 2010 [30,31]	DysportⓇ 100 U or 200 U.Repeat botulinum toxin type A injections and/or therapy were available at 3, 6, and 9 months if considered necessary after reassessment. location had no date.	4-week therapy program. The therapy program was provided by trained study therapists and each participant received 1 h per day, 2 times per week for 4 weeks. Stretch, positioning, passive/active, assisted upper limb activity, task-oriented practice	MAS, MI, MVG, NHPT, ARAT, pain, BI	3, 12 months	There was no significant difference in achievement of improved arm function (ARAT) at 1 month. Significant differences in favor of the intervention group were seen in muscle tone at 1 month; upper limb strength at 3 months; basic arm functional tasks (hand hygiene, facilitation of dressing) at 1, 3, and 12 months; and pain at 12 months.
Meythaler et al. 2009 [32]	BoNT-A 300U to 400U -location had no date.	Twice weekly 1-h sessions for each 12-week arm of the study (24 weeks total). Subjects did not begin treatment until 10 days from the injection.	Ashworth Scale, ROM, deep tendon reflex score, Grip strength, Pain, MAL, KB-ADL, BI, MOS-36	12, 24 weeks	Intervention group only improved the functional status of the subjects on the MAL Quality of Movement subscale and showed a trend toward significance in the Amount of Use subscale.
Lim et al. 2008 [33]	BotoxⓇ 100U by EMG -infraspinatus, subscapularis and pectoralis muscles	Physiotherapy during the 6-week period, a minimum of 2 visits per week	MAS, ROM, FMA, Pain, Physician global rating scale	2, 6, 12 weeks	No significant differences were observed between the 2 groups in terms of improvement in MAS, FMA or physician global rating.
Suputtitada et al. 2005 [34]	DysportⓇ by EMG 350 or 500 or1000 U - BB, FCU, FCR, FDP, FDS	Stretching, 3 days per week throughout the 6-month studied period	MAS, ARAT, VAS, BI	2, 4, 6, 8, 16, 24 weeks	The effect of functional disability was best at a dose of 500 U and the peak improvement was at week 8 after injection. A dose of 1000 U Dysport produced such an excess degree of muscle weakening that the number of randomized patients was reduced to five. BI and ARAT of all patients were decrease after injection.
Burbaud et al. 1996 [35]	DysportⓇ by EMG 1000U-Gastrocnemius, Soleus, TP, FDL	Active physiotherapy	MAS, FMA, Gait speed	30, 90, 120 days	Gait velocity was slightly but not significantly improved after BoNT-A injections.
Combined ES, FES and rehabilitation					
Fujita et al. 2018 [36]	BotoxⓇ by US 300U -Gastro Soleus TP FDL FPL	Physical therapy was performed for 2 weeks (two 1-h sessions per day). Stretch, leg resistance exercises, low-frequency electrical stimulation, electromyographic feedback, walking exercises	MAS, Clonus score, ROM, Gait speed	2 weeks	Intervention group was significantly changed in gait speed. For those who received BoNT-A+PT, biceps femoris activity and knee co-activation index during the loading response and tibialis anterior activity during the pre-swing phases increased, whereas soleus and rectus femoris activities during the swing phase decreased 2 weeks after the intervention.
Weber et al. 2010 [37]	OnabotulinumtoxinA -PrT, FCR, FCU, FDS, FDP, FPL	FES(1hour) and task practice therapy(1hour) included instructions for the home exercise program, and instruction on how to complete a daily patient exercise diary. 12 weeks	MAL, ARAT, MAL-Self-Report	6, 12 weeks	MAL-Observation mean item scores improved significantly from baseline to week-6, but did not remain significant at week-12. ARAT total scores also improved significantly from baseline to week-6 and were sustained at week-12. However, there were no significant differences between the FES and No-FES groups for any outcome variable over time.
Johnson et al. 2002, 2004 [38,39]	DysportⓇ 800U byEMG -Gastrocnemius, TP	A minimum of 3 sessions per week and outpatients 2 sessions per week	Walking speed, PCI, The Rivermead Motor Assessment, SF-36	2, 4, 6, 8, 12 weeks	Comparison of median walking speed (nonstimulated) in the control group with median stimulated walking speed shows a significant upward trend, the trend lines being significantly different in location.
Combined Robot and rehabilitation					
Erbil et al. 2018 [40]	BoNT-A by ES -Dosage and location had no data.	30 min of RAT plus 60 min of physical therapy, whereas controls received 90 min of physical therapy for 3 weeks during weekdays	MAS, Tardieu Scale, TUG, BBS, Rivermead Visual Gait Assessment	6, 12 weeks	Significant improvements were determined in both RAT and control groups regarding spasticity, balance, and gait functions after treatment. However, at post-treatment Weeks 6 and 12, change from baseline TUG, BBS, and Rivermead Visual Gait Assessment were significantly higher in RAT group than those of the control group.
Picelli et al. 2016 [41]	AbobotulinumtoxinA by US 750U -Gastrocnemius, Soleus	RAGT (30 min a day for five consecutive days) Immediately after BoNT‑A administration, all patients included in this study received a 60-min session of electrical stimulation of the injected muscles.	MAS, Tardieu Scale, 6MWT	1 month	No difference was found between groups as to MAS and the Tardieu scale measured at the affected ankle one month after BoNT-A. A significant difference in 6MWT was noted between groups at the post‑treatment evaluation.
Pennati et al. 2015 [42]	DysportⓇ by ES -pectoral, BB, TB, flexor carpi, FDS, FDP, FPL -Dosage had no data.	ReoGo system (10 sessions lasting 60 min each, 2 or 3 days a week)	MAS, FMA, Box&Block test, FIM, Euro-Qol	The end of robotic training	Both groups showed improvement in FMA. Higher improvement in Box&Block test was detected in Control group. MAS was improved more in Intervention group. In both groups, sEMG showed a reduction of co-contractions and an increase of agonist muscles recruitment during the reaching movement and the robotic exercises.
Combined Taping, Casting and rehabilitation					
Carda S et al. 2011 [43]	XeominⓇ by ES -each muscle 50-140U -Gastrocnemius, Soleus	After the first week all patients, irrespective of the allocation arm, underwent 30 min of gait training and 20 min of plantar flexor muscle stretching each day for one week under the guidance of a senior physical therapist.	MAS, ROM, Strength of ankle dorsal flexor muscles, 6MWT, 10MWT, Functional Ambulation Categories	20,90 days	Intervention group showed better and longer lasting results than with Control group
Karadag-Saygi E et al. 2010 [44]	BotoxⓇ by ES 150U-200U -Gastrocnemius	Active-assistive range of motion and stretching exercises were given as a home exercise program to both groups. Exercises were assigned twice daily for 20 min for 4 weeks	MAS, ROM, Gait velocity, Step length	2 weeks, 1, 3, 6 months	Improvement was recorded in both groups for all outcome variables. No significant difference was found between groups other than ROM, which was found to have increased more in control group at 2 weeks.
Combined CIMT					
Sun et al. 2010 [45]	DysportⓇ 1000U -BB, FDS, FDP, FCU, FCR	2 h/day, 3 days/week for 3 months	MAS, ARAT, MAL, Patients’ global satisfaction	4 weeks, 3, 6 months	Spasticity significantly improved in all subjects at 4 weeks and 3 months postinjection without between-group differences. Intervention group showed significantly greater improvements in elbow, wrist, and finger spasticity, affected upper extremity real-world arm function and laboratory motor activity than the control group at 6-month postinjection.

Abbreviations: B, brachialis; BB, biceps brachii; BR, brachioradialis; EPL, extensor pollicis longus; FCR, flexor carpi radialis; FCU, flexor carpi ulnaris; FDB, flexor digitorum brevis; FDL, flexor digitorum longus; FDP, flexor digitorum profundus; FDS, flexor digitorum superficialis; FPL, flexor pollicis longus; PrT, pronator teres; RF, rectus femoris; TB, triceps brachii; TP, tibialis posterior; 10MWT, 10 m walk test; 6MWT, six minute walk test; ADL, activities of daily living; ArmA, the arm activity measure; BBS, Berg balance scale; BoNT-A, botulinum toxin A; CSI, clinic spasticity influx; EMG, electromyography; ES, electrical Stimulation; FIM, functional independence measure; FMA, Fugl–Meyer Assessment; GAS, goal attainment scaling; MAS, Modified Ashworth Scale; MRc, The medical research council scale; OT, occupation therapy; PT, physical therapy; ROM, range of motion; TUG, Timed Up and Go Test; US, ultrasound; ARAT, Action Research Arm Test; BI, Barthel Index; KB-ADL, Klein Bell Activity of Daily Living Scale; MAL, the motor activity log; MI, Motricity Index; MVG, maximum voluntary grip strength; NHPT, Nine Hole Peg Test; REPAS, resistance to passive movement scale; VAS, visual analogue scale; WMFT, Wolf Motor Function Test; FES, functional electrical stimulation; PCI, physiological cost index; QoL, assessment of quality of life; RAGT, robot-assisted gait training; RAT, robot-assisted training; and CIMT, constraint-induced movement therapy.

**Table 3:** List of control groups.

Control Group	Study
BoNT-A only	Devier et al. 2017 [21], Roche et al. 2015 [22], Fujita et al. 2018 [36], Picelli et al. 2016 [41]
Placebo injection	Prazeres A et al. 2018 [19], Tao et al. 2015 [24], Rosales et al. 2012 [27], Wolf et al. 2012 [29], Meythaler et al. 2009 [32]
	Suputtitada et al. 2005 [34], Burbaud et al. 1996 [35]
Rehabilitation only	Umar et al. 2018 [20], Ding et al. 2015 [23], Demetrios et al. 2014 [25], Hesse et al. 2012 [28], Shaw et al. 2011, 2010 [30,31]
	Johnson et al. 2002, 2004 [38,39], Pennati et al. 2015 [42], Carda S et al. 2011 [43], Karadag-Saygi E et al. 2010 [44]
BoNT-A + Rehabilitation	Weber et al. 2010 [37], Erbil et al. 2018 [40], Sun et al. 2010 [45]
Other	Uchiyama Y et al. 2018 [18], Pimentel et al. 2014 [26], Lim et al. 2008 [33]

**Table 4:** Risk of bias summary.

Risk of Bias	Random Sequence Generation	Allocation Concealment	Blinding of Participants and Personnel	Blinding of Outcome Assessment	Incomplete Outcome Data	Selective Reporting	Other Bias
Uchiyama Y et al. 2018 [18]	High	High	High	High	Low	Low	High
Prazeres A et al. 2018 [19]	Low	Low	Low	Low	Low	Unclear	High
Umar et al. 2018 [20]	Low	Low	High	Low	Low	Low	Low
Devier D et al. 2017 [21]	Low	Low	High	Low	Low	High	High
Roche N et al. 2015 [22]	High	Unclear	High	High	Unclear	Low	Low
Ding XD et al. 2015 [23]	High	Unclear	High	High	Unclear	Low	High
Tao et al. 2015 [24]	Unclear	Unclear	Low	Low	Low	Low	Low
Demetrios M et al. 2014 [25]	High	High	High	Low	Low	High	High
Pimentel LH et al. 2014 [26]	Unclear	Unclear	Low	Low	Low	Low	Unclear
Rosales RL et al. 2012 [27]	Low	Low	Low	Low	Low	High	High
Hesse S et al. 2012 [28]	Low	High	High	High	Low	Low	Low
Wolf SL et al. 2012 [29]	Low	Low	Low	Low	Low	High	Low
Shaw LC et al. 2011, 2010 [30,31]	Low	Low	High	High	Low	Low	High
Meythaler JM et al. 2009 [32]	Unclear	Low	Low	Low	Low	High	High
Lim JY et al. 2008 [33]	Low	Low	Low	Low	High	High	Low
Suputtitada et al. 2005 [34]	Low	High	Low	Low	Low	High	High
Burbaud et al. 1996 [35]	Unclear	Unclear	Low	Unclear	Low	High	High
Fujita K 2018 [36]	High	High	High	High	Low	Low	High
Weber D.J. et al. 2010 [37]	Low	High	High	Low	Low	Low	High
Johnson CA et al. 2002, 2004 [38,39]	Low	High	High	High	Low	High	High
Erbil D et al. 2018 [40]	Unclear	High	High	High	Low	Low	High
Picelli A et al. 2016 [41]	Low	Low	High	Low	Low	Low	Low
Pennati G.V. et al. 2015 [42]	Low	Low	High	Low	Low	High	High
Carda S et al. 2011 [43]	Low	High	High	Low	Low	Low	Low
Karadag-Saygi E et al. 2010 [44]	Unclear	High	High	Low	Low	Low	Low
Sun SF et al. 2010 [45]	Low	High	High	Low	Low	Low	Low

## Appendix A

((“stroke”[MeSH Terms] OR “stroke”[All Fields]) OR (“stroke”[MeSH Terms] OR “stroke”[All Fields] OR (“cerebral”[All Fields] AND “vascular”[All Fields] AND “accident”[All Fields]) OR “cerebral vascular accident”[All Fields]) OR ((“ischemia”[MeSH Terms] OR “ischemia”[All Fields] OR “ischemic”[All Fields]) AND (“stroke”[MeSH Terms] OR “stroke”[All Fields])) OR (“intracranial hemorrhages”[MeSH Terms] OR (“intracranial”[All Fields] AND “hemorrhages”[All Fields]) OR “intracranial hemorrhages”[All Fields] OR (“hemorrhagic”[All Fields] AND “stroke”[All Fields]) OR “hemorrhagic stroke”[All Fields])) AND ((“botulinum toxins”[MeSH Terms] OR (“botulinum”[All Fields] AND “toxins”[All Fields]) OR “botulinum toxins”[All Fields] OR (“botulinum”[All Fields] AND “toxin”[All Fields]) OR “botulinum toxin”[All Fields]) OR ((“botulinum toxins”[MeSH Terms] OR (“botulinum”[All Fields] AND “toxins”[All Fields]) OR “botulinum toxins”[All Fields] OR (“botulinum”[All Fields] AND “toxin”[All Fields]) OR “botulinum toxin”[All Fields]) AND (“therapy”[Subheading] OR “therapy”[All Fields] OR “therapeutics”[MeSH Terms] OR “therapeutics”[All Fields])) OR (Antispastic[All Fields] AND (“therapy”[Subheading] OR “therapy”[All Fields] OR “therapeutics”[MeSH Terms] OR “therapeutics”[All Fields]))) AND ((“rehabilitation”[Subheading] OR “rehabilitation”[All Fields] OR “rehabilitation”[MeSH Terms]) OR (“physical therapy modalities”[MeSH Terms] OR (“physical”[All Fields] AND “therapy”[All Fields] AND “modalities”[All Fields]) OR “physical therapy modalities”[All Fields] OR (“physical”[All Fields] AND “therapy”[All Fields]) OR “physical therapy”[All Fields]) OR (“occupational therapy”[MeSH Terms] OR (“occupational”[All Fields] AND “therapy”[All Fields]) OR “occupational therapy”[All Fields]) OR (Intensive[All Fields] AND (“rehabilitation”[Subheading] OR “rehabilitation”[All Fields] OR “rehabilitation”[MeSH Terms])) OR ((“interdisciplinary studies”[MeSH Terms] OR (“interdisciplinary”[All Fields] AND “studies”[All Fields]) OR “interdisciplinary studies”[All Fields] OR “multidisciplinary”[All Fields]) AND (“rehabilitation”[Subheading] OR “rehabilitation”[All Fields] OR “rehabilitation”[MeSH Terms]))) AND ((“physiology”[Subheading] OR “physiology”[All Fields] OR “function”[All Fields] OR “physiology”[MeSH Terms] OR “function”[All Fields]) OR (“aptitude”[MeSH Terms] OR “aptitude”[All Fields] OR “ability”[All Fields]) OR (“walking”[MeSH Terms] OR “walking”[All Fields] OR “walk”[All Fields]) OR “capacity”[All Fields]).
